# Endemic Lineages of *Batrachochytrium dendrobatidis* Are Associated With Reduced Chytridiomycosis-Induced Mortality in Amphibians: Evidence From a Meta-Analysis of Experimental Infection Studies

**DOI:** 10.3389/fvets.2022.756686

**Published:** 2022-03-04

**Authors:** Anat M. Belasen, Imani D. Russell, Kelly R. Zamudio, Molly C. Bletz

**Affiliations:** ^1^Department of Ecology and Evolutionary Biology, Cornell University, Ithaca, NY, United States; ^2^Society for Conservation Biology, Washington, DC, United States; ^3^Department of Integrative Biology, University of Texas at Austin, Austin, TX, United States; ^4^Department of Ecology, Evolution, and Marine Biology, University of California-Santa Barbara, Santa Barbara, CA, United States; ^5^Department of Biology, University of Massachusetts-Boston, Boston, MA, United States

**Keywords:** *Batrachochytrium dendrobatidis* (Bd), meta-analysis, pathogen genotypes, experimental infection, amphibian, virulence, chytridiomycosis

## Abstract

Emerging infectious wildlife diseases have caused devastating declines, particularly when pathogens have been introduced in naïve host populations. The outcome of disease emergence in any host population will be dictated by a series of factors including pathogen virulence, host susceptibility, and prior opportunity for coevolution between hosts and pathogens. Historical coevolution can lead to increased resistance in hosts and/or reduced virulence in endemic pathogens that allows stable persistence of host and pathogen populations. Adaptive coevolution may also occur on relatively short time scales following introduction of a novel pathogen. Here, we performed a meta-analysis of multi-strain *Batrachochytrium dendrobatidis* (Bd) infection experiments to test whether: (1) amphibian hosts exhibit lower mortality rates when infected with strains belonging to endemic Bd lineages relative to the Global Panzootic Lineage (Bd-GPL), hypothetically owing to long co-evolutionary histories between endemic Bd lineages and their amphibian hosts; and (2) amphibians exhibit lower mortality rates when infected with local Bd-GPL strains compared with non-local Bd-GPL strains, hypothetically owing to recent selection for tolerance or resistance to local Bd-GPL strains. We found that in a majority of cases, amphibians in endemic Bd treatments experienced reduced mortality relative to those in Bd-GPL treatments. Hosts presumed to have historically coexisted with endemic Bd did not show reduced mortality to Bd-GPL compared with hosts that have not historically coexisted with endemic Bd. Finally, we detected no overall difference in amphibian mortality between local and non-local Bd-GPL treatments. Taken together, our results suggest that long-term historical coexistence is associated with less disease-induced mortality potentially due to hypovirulence in endemic Bd lineages, and that more recent coexistence between amphibians and Bd-GPL has not yet resulted in reduced host susceptibility or pathogen virulence. This corroborates previous findings that Bd-GPL introduced via the global amphibian trade has a high capacity for causing disease-induced mortality.

## Introduction

Emerging wildlife diseases pose significant threats to global biodiversity, but the mechanisms by which some hosts are able to survive recently emerged diseases are not yet fully understood (1). To aid in assessment of disease risk in host populations and species of concern, it is critical to understand the mechanisms that determine the outcome of infection. Hosts may be *susceptible* to infection and/or disease-induced mortality, or may survive infection due to *resistance* (i.e., the ability to prevent infection or reduce infection load) and/or *tolerance* (i.e., the ability to sustain infection without experiencing substantial negative impacts). Likewise, infection outcomes can also depend on variation in pathogen virulence, which can result from genotypic differences among pathogen strains or genotypes (2–4). While contemporary conditions (i.e., local abiotic and biotic environments) may significantly influence host susceptibility [e.g., (5)] and/or pathogen virulence [reviewed in Turner et al. (6)], prior interactions between host and pathogen populations may also contribute to significant variability in disease outcomes. For example, a pathogen introduced to naïve host populations may cause severe outbreaks (i.e., epizootic disease), while recently or historically co-occurring hosts and pathogens may show disease dynamics that are tempered with mild or no disease despite pathogen presence [i.e., enzootic disease or absent disease; (7, 8)]. When the co-evolutionary trajectory between host and pathogen takes the form of a classical oscillating arms race between host resistance and pathogen virulence, different host and pathogen populations may exhibit different levels of resistance and virulence at the same point in time (9). In these cases, current infection outcomes can vary according to host-pathogen co-evolutionary history, leading to a “geographic mosaic” of disease threat (10).

Amphibian chytridiomycosis caused by *Batrachochytrium dendrobatidis* (Bd) is one of the most devastating wildlife diseases documented in the scientific literature to date (11–13). Bd is believed to have originated in East Asia (14), and one hypervirulent lineage, the Global Panzootic Lineage (hereafter Bd-GPL) spread globally by the transcontinental amphibian trade (15). Bd-GPL has been implicated in die-offs around the world (12), and the prevailing hypothesis is that introductions of Bd-GPL were particularly damaging in naïve amphibian communities where many or all hosts lacked any effective form of Bd resistance (16, 17). In East Asia, Brazil, South Africa, and Switzerland, additional geographically restricted and early-diverged genotypic lineages of Bd have been identified that may have much longer coevolutionary histories with amphibians compared to Bd-GPL (14). These lineages named *Bd*-Asia, *Bd*-Brazil, *Bd*-Cape, and *Bd*-CH, are referred to as endemic Bd lineages. Studies have shown that at least some endemic Bd lineages were associated with low mortality rates in susceptible species (14) and that amphibian species that have coexisted historically with endemic Bd lineages appear to exhibit Bd resistance and/or tolerance [e.g. (18)]. These studies support the hypotheses that due to a long history of host-pathogen coevolution, endemic Bd lineages are broadly hypovirulent, and communities of amphibians in Bd-endemic areas could be protected from Bd outbreaks (16, 19).

However, as studies of Bd have increased, we have found surprising diversity in Bd lineages and their distributions, and also unpredicted variability in disease outcomes. Experimental results have varied widely according to focal host: for instance, initial experiments did not use hosts that had locally adapted to presumed hypovirulent genotypes [e.g. (14)], and later experiments showed host species-level variation in disease susceptibility even when infected by the same endemic Bd lineage (18). Retrospective studies of Bd infections in museum specimens revealed that Bd-GPL may have been present for longer than previously thought (i.e., predating amphibian trade) in areas such as southeastern Brazil (20) and the United States (21, 22), providing the opportunity for local adaptation to Bd-GPL. Furthermore, field studies have shown that multiple genotypes (endemic Bd and Bd-GPL) and multiple Bd-GPL strains can be present in sympatry in wild populations [e.g. (23)], with the potential to produce differing disease outcomes across amphibian communities. For example, local Bd-GPL strains, defined here as the only or predominant Bd-GPL strain in a host community, may protect hosts from more virulent non-local Bd-GPL strains found in surrounding communities (24). Given that in some cases Bd-GPL has been introduced through several independent events to disparate amphibian communities (25), we may expect variable levels of local, shorter-term co-adaptation between amphibian hosts and the Bd-GPL strains found in different host communities, climates, and geographies. Because Bd is globally distributed and is a highly generalist pathogen capable of infecting at least 500 amphibian species (12, 13), understanding how coevolution and potential co-adaptation of amphibians and Bd impact infection outcomes is critical to predicting and mitigating future chytridiomycosis declines.

Here, we perform meta-analyses of host mortality in multi-strain, controlled Bd infection experiments to determine whether opportunity for historical or recent selection alters disease outcomes in amphibian hosts that are susceptible to disease-induced mortality. Multiple factors may contribute to experimental disease outcomes, including characteristics of the host source population; therefore we restricted our meta-analysis to controlled experiments and calculated pairwise effect sizes for Bd infection treatments involving hosts from the same source population or locality. This approach allows us to summarize the impact of Bd infection across experimental studies published to date using comparable quantitative effect sizes. We test two hypotheses: (1) the historical adaptation (HA) hypothesis posits that endemic Bd lineages show hypovirulence relative to Bd-GPL as a result of long coevolutionary histories with their amphibian hosts; and (2) the recent adaptation (RA) hypothesis posits that amphibians show lower susceptibility to local Bd-GPL strains relative to non-local Bd-GPL strains due to recent and rapid adaptation or co-adaptation of amphibian hosts and Bd. To test these hypotheses, we performed two meta-analyses to evaluate whether Bd genotype and/or locality impacts mortality due to standardized Bd infection. First, to test the HA hypothesis, we analyzed the effect of Bd genotypic lineage to determine whether endemic Bd strains result in lower mortality than Bd-GPL strains, regardless of host and Bd origin (i.e., collection locality). Second, to test the RA hypothesis, we analyzed the effect of recent geographic co-occurrence of host and pathogen to determine whether local Bd-GPL strains (collected from the same locality as the focal host) result in lower mortality relative to non-local Bd-GPL strains. Our results provide insight into the significance of host-pathogen adaptation at two temporal scales in chytridiomycosis outcomes, and suggest future directions for research that would aid conservation in the face of devastating wildlife disease.

## Methods

### Data Compilation

To test the HA and RA hypotheses, we performed two meta-analyses of experimental Bd studies on susceptible amphibians, here defined as hosts that can experience chytridiomycosis-induced mortality. As a starting point for compiling experimental Bd studies, we used the systematic review of experimental studies performed by Blaustein et al. (26). This study utilized a Web of Science search supplemented with a Google Scholar search for studies between 1999 and 2017 using the search terms “*Batrachochytrium dendrobatidis* + amphibians + experiments (26).” We retained 14 studies identified by Blaustein et al. (26) that involved multi-strain infection experiments. We replicated this search to compile recent studies published between 2017 and 2021 using Web of Science (searched on April 21, 2021), supplemented with a Google Scholar search [October 7, 2021 and collated in Publish or Perish (27)], and included one unpublished study from the authorship team's collaborator (28).

Our supplemental search resulted in 3,177 publications total, of which four met our criteria of being multi-strain experiments that reported mortality in susceptible host species (i.e., species that exhibited mortality in at least one infection treatment; Supplementary Figure 1). Study units were excluded from analyses if: (i) mortality in control treatments significantly exceeded mortality in experimental infection treatments; (ii) Bd genotype (i.e., belonging to GPL or an endemic lineage) and/or collection locality information were not available; and/or (iii) hosts were fully resistant to chytridiomycosis (i.e., experienced zero mortality in all treatments). Study units were defined as a single study x species x treatment combination.

**Figure 1:**
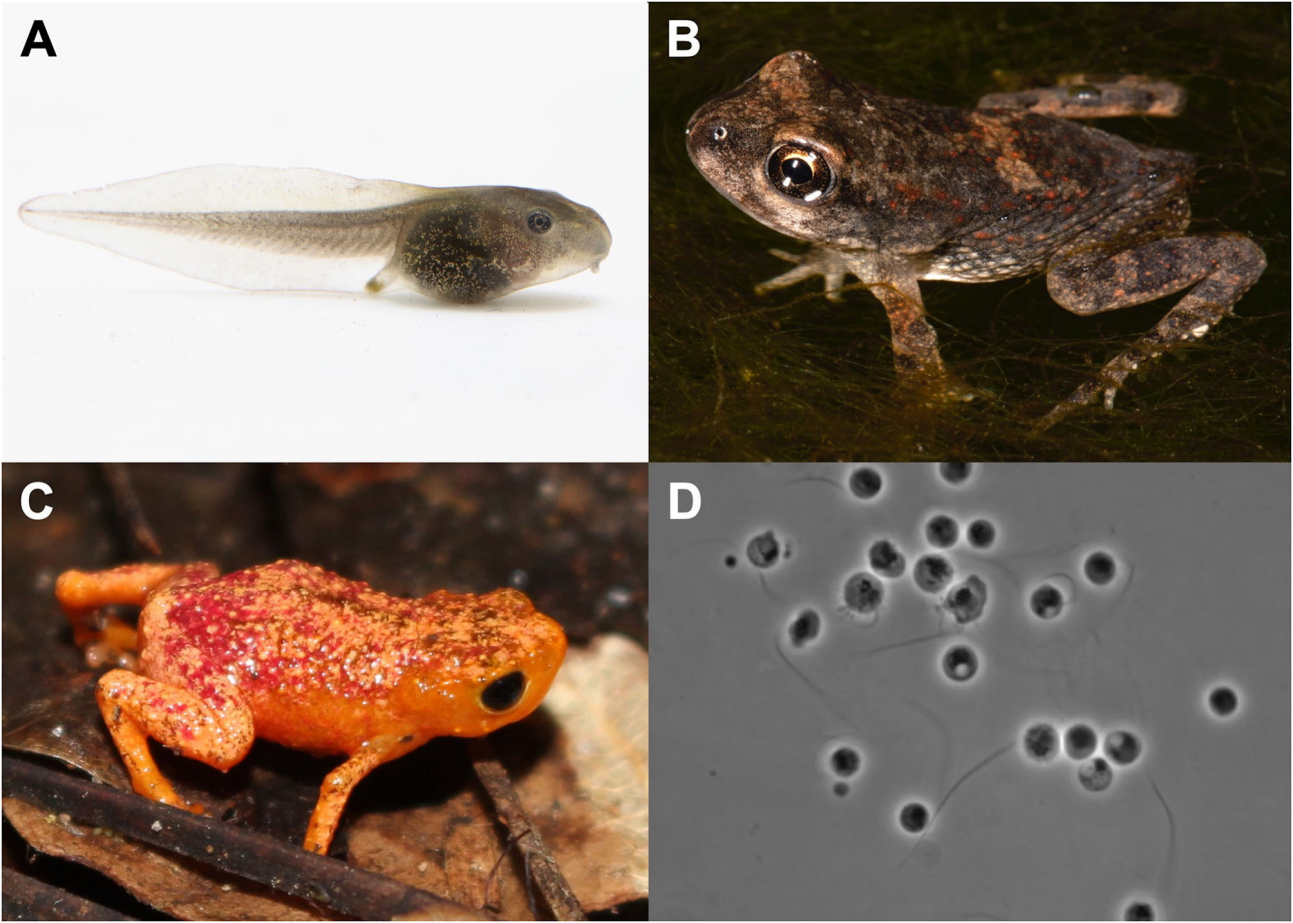
Select study organisms included in the meta-analysis. **(A)** *Rana sylvatica* (wood frog) larva. This is one of three species for which data were available for larval experimental infections. Photo by Brian Gratwicke. **(B)** Bufonid metamorph. Recently metamorphosed amphibians are believed to be particularly susceptible to chytridiomycosis. Photo by Todd Pierson. **(C)** *Brachycephalus pitanga* (pumpkin toadlet) is a tiny terrestrial direct developer and is endemic to Brazil's Atlantic Forest. Photo by Anat Belasen. **(D)** *Batrachochytrium dendrobatidis* zoospores on 1%T visualized under a stereoscope. Photo by Joyce Longcore.

Between Blaustein et al. (26) and our supplemental search, we recovered 30 study units across 12 publications, covering 19 ecologically diverse species of amphibians and three amphibian life stages (Figure 1 and Supplementary Figure 1). Studies used in the HA analysis (*n* = 21 study units, Table 1) included either wild-collected or captive-reared amphibians, and were required to include at least two genotypes of Bd (Bd-GPL plus one endemic genotype, i.e., Bd-Brazil, Bd-Asia, Bd-Cape, or Bd-CH) that were separately inoculated on test animals. If experimental Bd strains were not genotyped by the authors of the study, genotype information was sourced from papers that genotyped these strains using DNA sequencing and phylogenetic analyses (Supplementary Table 1). In one case, Bd genotype was inferred by the current authors to be Bd-GPL [isolate LC63 from a captive Australian frog (38)]. Studies used in the RA analysis (*n* = 17 study units, Table 1) were required to include wild-collected amphibians and at least two strains of Bd-GPL, at least one of which was collected locally (where focal hosts were collected) and at least one of which was collected non-locally.

**Table 1:** Study units included in the two meta-analyses.

	**Host species**	**Study number**	**Life stage at Bd exposure**	**Exposure dose**	**Experiment duration**	**Endemic Bd used**	**Historical Bd coexistence**	**Citation**
Historical Adaptation (HA) Analysis	*Alytes muletensis*	*1*	larva*†	Cumulative: 2.3 k zsp over 2w	160 days	Bd-Cape	Yes	Doddington et al. (29)
	*Bombina orientalis*	*6a*	adult	8 × 105 zsp	3 months	Bd-Asia1	Yes	Fu and Waldman (18)
	*Brachycephalus ephippium*	*7a*	adult	1.8 × 106 zsp	60 days	Bd-Brazil	Yes	Greenspan et al. (30)
	*Brachycephalus pitanga*	*9*	adult	3.9 × 106 zsp	15 days	Bd-Brazil	Yes	McDonald (28)
	*Bufo bufo*	*2a-b*	larva*†	Cumulative: 190 or 19k zsp every 4d over 2w (treatments averaged for analysis) Max total−5.7 × 10^4^ zsp	80 days	Bd-Cape	No	Fisher et al. (2)
		*3a-b*	larva*	Cumulative: 3–17 k zsp every 4d × 8 reps; Max total−1.36 × 10^5^ zsp	122 days	Bd-Cape	No	Farrer et al. (31)
		*4a-c*	larva*^†^	Cumulative: 7.5–37.5 k zsp every 4d × 8 reps; Max total = 3 × 10^5^	42+ days	Bd-Asia1, Bd-Cape, Bd-CH	No	O'Hanlon et al. (14)
		*4d-f*	metamorph	Cumulative: 10–36 k zsp every 4 d × 5 reps; Max total = 8.5 × 10^4^ zsp	22 days	Bd-Asia1, Bd-Cape, Bd-CH	No	O'Hanlon et al. (14)
	*Dendropsophus minutus*	*7b*	adult	1.8 × 106 zsp	60 days	Bd-Brazil	Yes	Greenspan et al. (30)
	*Hymenochirus curtipes*	*8*	adult	1.25 × 106 zsp	25 days	Bd-Brazil	No	Jenkinson et al. (32)
	*Ischnocnema parva*	*7c*	adult	1.8 × 106 zsp	60 days	Bd-Brazil	Yes	Greenspan et al. (30)
	*Rana sylvatica*	*5a*	larva*	106 zsp/tank at 0 and 17 d	70 days	Bd-Brazil	No	Becker et al. (4)
	*Litoria caerulea*	*6b*	adult	5 × 105 zsp	3 months	Bd-Asia1	No	Fu and Waldman (18)
Recent Adaptation (RA) Analysis	*Alytes obstetricans*	*10a-d*	metamorph	1 × 10^∧^6 fo 24 h	13 weeks			Greener et al. (24)
	*Anaxyrus americanus*	*11a-c*	metamorph	3.33 × 105 zsp	73 days			Burrow et al. (33)
		*12a*	metamorph	10^6−7^ zsp + 10^5−6^ spg	40 days			Gahl et al. (34)
	*Anaxyrus boreas*	*13a-b*	larva^†^	105 zsp	20 days			Dang et al. (35)
	*Bufo bufo*	*2c*	larva*^†^	Cumulative: 190 or 19 k zsp every 4 d over 2 w (treatments averaged for analysis)	80 days			Fisher et al. (2)
		*14*	metamorph	4 ml of 4 × 10^∧^6 zsp/ml inoculum for consecutive days	30 days			Meurling et al. (36)
	*Physalaemus fernandezae*	*15*	metamorph	6 × 10^∧^4 for 5 h	14 days			Arellano et al. (37)
	*Pseudacris regilla*	*13c-d*	larva^†^	105 zsp	20 days			Dang et al. (35)
	*Rana clamitans*	*12b*	metamorph	10^6−7^ zsp + 10^5−6^ spg	40 days			Gahl et al. (34)
	*Rana pipiens*	*12c*	metamorph	10^6−7^ zsp + 10^5−6^ spg	40 days			Gahl et al. (34)
	*Rana sylvatica*	*12d*	larva*^†^	10^6−7^ zsp + 10^5−6^ spg	40 days			Gahl et al. (34)
		*5b*	larva*	106 zsp/tank	70 days			Becker et al. (4)
	*Rana cascadae*	*13e-f*	larva^†^	105 zsp	20 days			Dang et al. (35)
	*Rana onca*	*16a-b*	larva* & metamorph	3 × 106 zsp over 3d	18 weeks			Waddle et al. (38)

**Figure 2:**
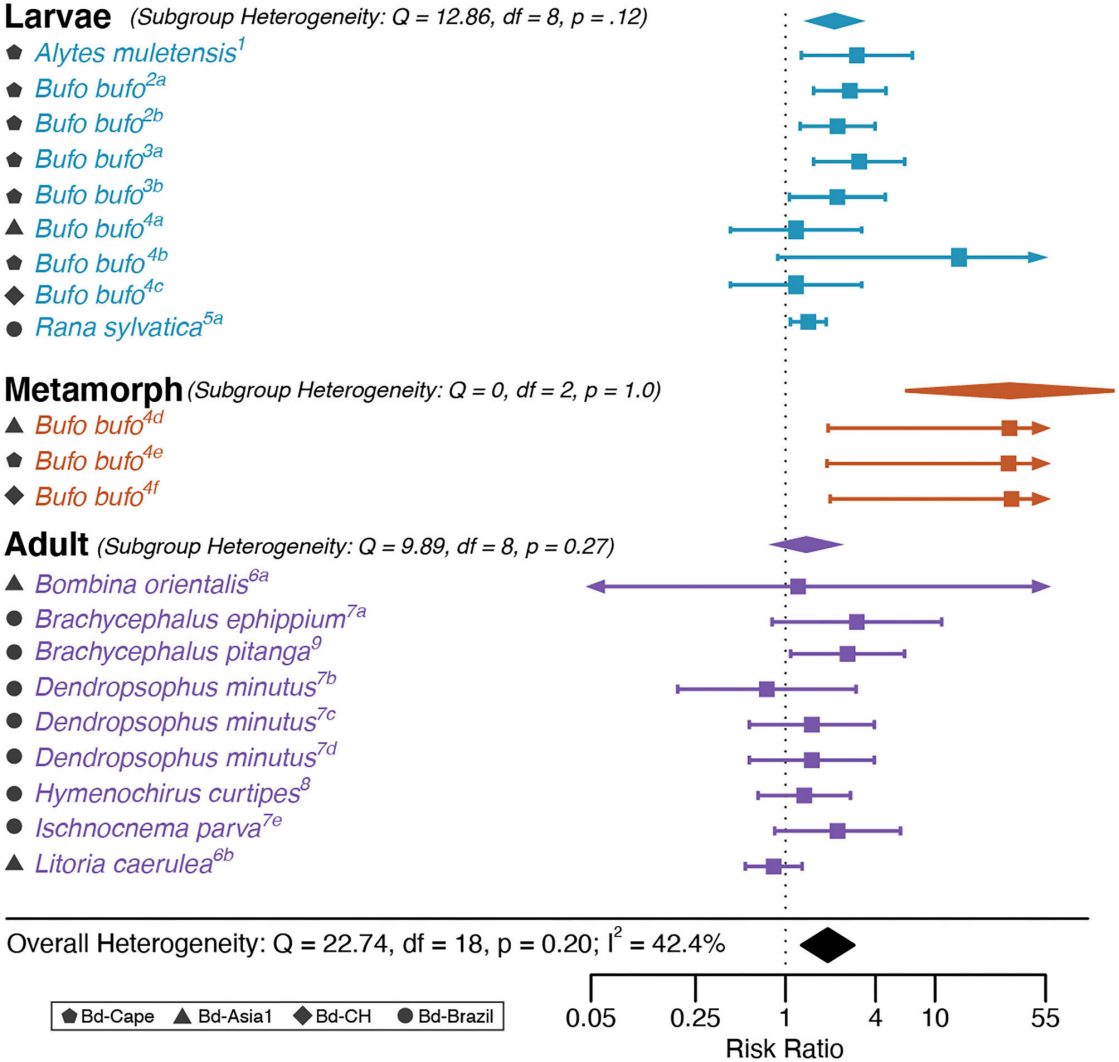
Historical Adaptation analysis forest plot depicting Risk Ratio effect sizes for each life stage category. Squares indicate mean effect size for each study unit and error bars depict 95% Confidence Interval (CI). The meta-analytic mean effect size across study units (black diamond) was calculated without any subgroup moderator terms. Arrows on error bars indicate that the CI exceeds the provided x-axis scale. Study unit ID numbers correspond to Table 1. Heterogeneity test statistics (Q), degrees of freedom (df), and *p*-values for Cochran's Q test of heterogeneity are reported for each subgroup and the overall model.

Studies in the top half of the table were included in the Historical Adaptation (HA) analysis, while those in the bottom half were included in the Recent Adaptation (RA) analysis. Study number corresponds with superscript notations in Figures 2, 4. In studies where hosts were exposed to Bd as larvae, asterisks (*) denote that mortality values were reported post-metamorphosis, and crosses () denote that mortality values were reported pre-metamorphosis. Use of both symbols denotes mortality was recorded across both life stages. Abbreviations in the exposure dose column are as follows: zsp, zoospore; spg, zoosporangia; d, days; w, weeks; reps, repetitions. For HA analysis studies, the endemic Bd treatment column indicates the genotype of the endemic Bd lineage used in the experiment. Historical Bd coexistence is reported based on known or hypothesized historical coexistence of the focal host with the endemic Bd used in the experiment. Citation is provided with corresponding literature cited reference number in parentheses. See Supplementary Table 1 for strain-specific genotyping information.

### Data Analysis and Visualization

Data extraction, analyses, and visualizations were performed in the R environment (39). For each study unit, we extracted the sample sizes for control and experimental infection treatments, and percent mortality for each genotype/strain of Bd. If mortality values were not directly present in publication text, we used the metaDigitise package in R (40) to extract numeric data from published survival curves. Mortality values were averaged in cases where there were multiple isolates from a given genotype x locality combination tested on the same amphibian host species in different treatments within a given study. We also compiled metadata for each study unit, including host adult ecology, host reproductive mode, host life stage at infection, and historical coexistence status with endemic Bd (i.e., whether the focal host species historically coexisted with the endemic Bd lineage used in the experiment).

In experiments using sensitive amphibian hosts, background mortality can be common. While we excluded studies that contained significantly higher mortality in controls relative to infection treatments, we included 24 study units with low levels of control mortality that did not significantly exceed treatment mortality. In these cases, the control mortality rate was subtracted from each treatment mortality rate to obtain a “background mortality-corrected” rate for each treatment. In cases where control mortality marginally exceeded treatment mortality (*n* = 3 studies in the HA analysis, *n* = 1 study in the RA analysis), the corrected treatment mortality value was rounded up to zero. In all of these studies, mortality was significantly higher in at least one infection treatment than in the control treatment, indicating Bd-induced mortality above potential background mortality due to captive conditions.

Unless otherwise stated, all calculations and analyses were performed in the R metafor package (41). To estimate the effect size of Bd genotype (HA analysis) or source locality (RA analysis) on mortality, log Risk Ratio was calculated for each study unit. Subsequently, linear mixed models were constructed to analyze effect sizes weighted by variance for each analysis. Models constructed for each analysis were fitted using a restricted maximum likelihood (REML) approach. A generalized, weighted least squares extension of Cochran's Q test (41) and *I*^2^ (42) were calculated to test whether the heterogeneity among effect sizes was greater than expected. Outliers or influential cases were identified by calculating Cook's distances (≥0.45), Hat values [≥3^*^(1/k)], and dfbetas [≥1; (41, 43)], and removed if at least two metrics identified the outlier.

To test whether variation among effect sizes was associated with any subgroup variables, three models were constructed for each analysis: (1) intercept-only (base) models; (2) multifactor (full) models that included all subgroup terms (HA only); and (3) single-factor models that consisted of separate models for each subgroup term. Subgroup variables included life stage at infection (both HA and RA) and historical coexistence (HA only). Host adult ecology and host reproductive mode were not included as subgroup moderators due to a lack of sufficient statistical power (little or no replication for some factor levels). In all models (including base models), study (publication) ID was included as a random term to account for non-independence among study units within publications (44). AIC was used to determine model ranking within each analysis. Average log Risk Ratios were exponentiated to calculate the Risk Ratio values for subgroup terms that were significant in single-factor models. Fligner-Killeen non-parametric tests for homogeneity of variance were used to evaluate variance among subgroup variables of interest. Results were visualized in metafor (41) and ggplot2 (45).

## Results

### Historical Adaptation Analysis

In the HA analysis, Bd genotype was associated with a significantly positive log Risk Ratio according to the intercept-only model, indicating that Bd-GPL infections resulted in higher mortality relative to endemic lineages of Bd (RR = 1.79, *Z* = 3.99, *p* < 0.0001; Figure 2). The HA dataset showed a high degree of heterogeneity (*I*^2^ = 42.38 %; Q = 40.104, *p* = 0.0048). No outliers or influential cases were detected (Cook's D < 0.45; Hat < 0.14; dfbetas < 1).

The multifactor (full) HA model exhibited better fit than the intercept-only (base) model (AIC = 38.77 for the full model vs. 51.68 for the base model), and reduced heterogeneity in the dataset such that it was not significant (Q = 16.12, *p* = 0.516). The HA single-factor model that included host life stage was significant (QM = 14.24, *p* = 0.0008; Figure 2). Risk Ratio was higher in individuals infected as metamorphs compared with those infected as larvae or adults, and higher in individuals infected as larvae than as adults (RR_metamorph_ = 6.9, RR_larvae_ = 1.99.1, RR_adults_ =1.36; Figure 2).

**Figure 3:**
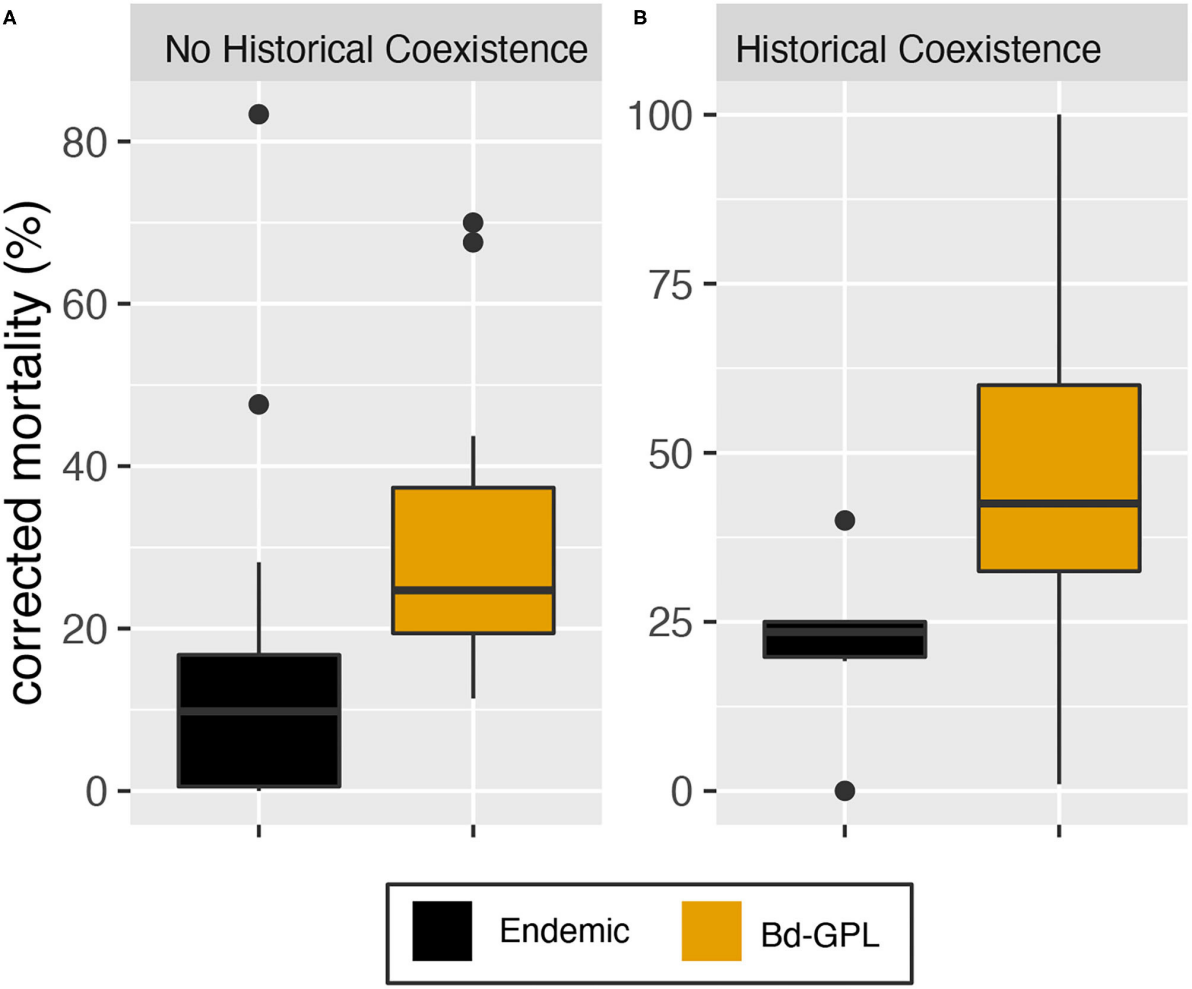
Mortality outcomes pooled by Bd lineage. **(A)** Mortality in species that have not historically coexisted with the endemic Bd used in the experiment. **(B)** Mortality in species that have historically coexisted with the endemic Bd used in the experiment. Mortality values have been corrected for background (control treatment) mortality. Endemic Bd-associated mortality is depicted in black and Bd-GPL-associated mortality is depicted in ochre.

The single-factor model for historical coexistence was not statistically significant. However, data visualization showed evidence of greater variability in mortality due to infection with endemic Bd among hosts that had not historically coexisted with endemic Bd (Figure 3 and Supplementary Figure 2). We further explored this relationship by testing for statistical differences in mortality variance in study units with historical coexistence vs. those without historical coexistence for endemic vs. panzootic (Bd-GPL) Bd genotypes. We found no statistically significant differences in variance for either Bd genotype group (Fligner-Killeen test: Bd-GPL–*X*^2^ = 1.57, *p* = 0.211; endemic Bd–*X*^2^ = 0.46, *p* = 0.498).

### Recent Adaptation Analysis

**Figure 4:**
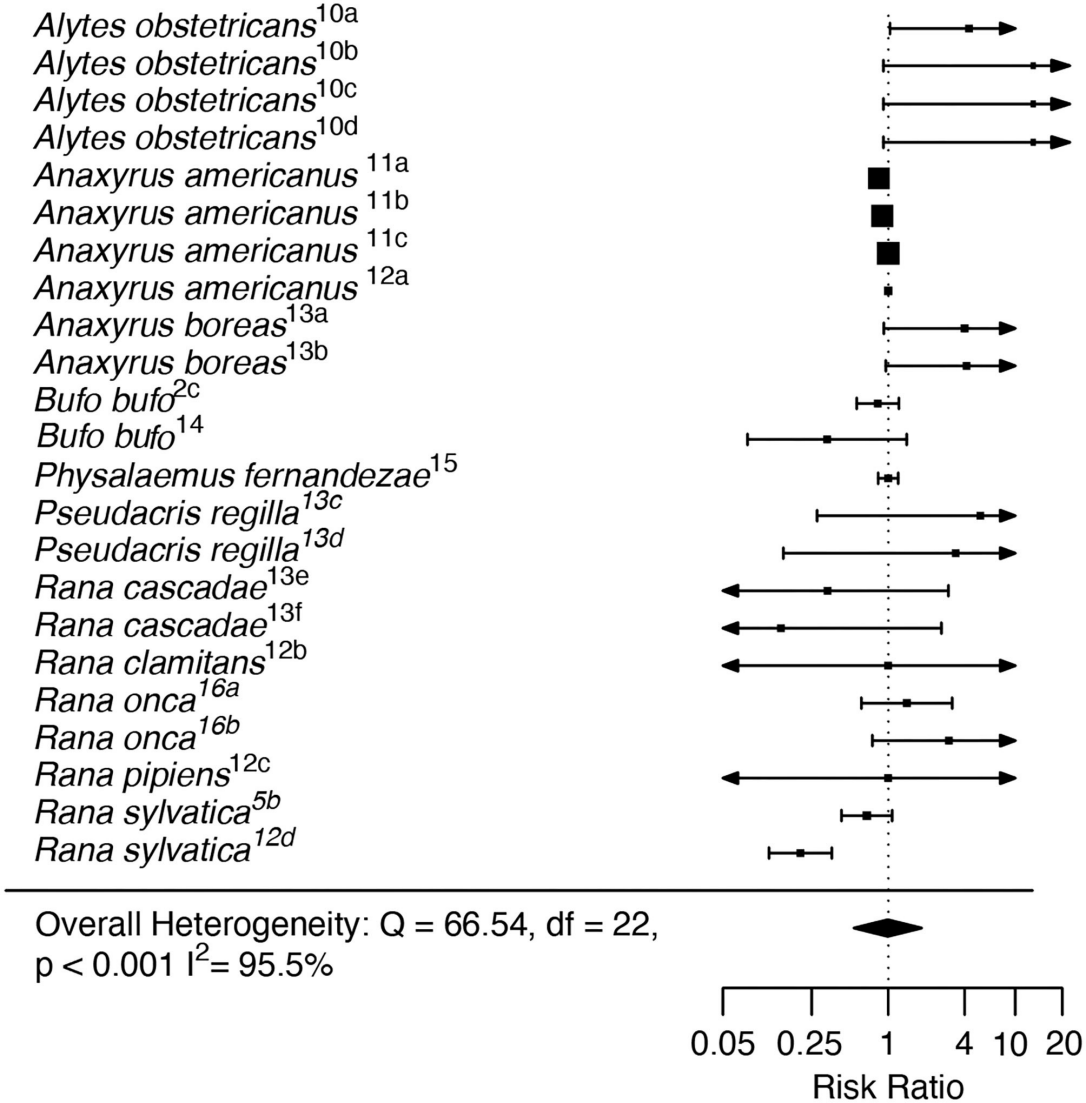
Recent Adaptation analysis forest plot depicting Risk Ratio effect sizes across study units. Squares indicate mean effect size for each study unit, and error bars depict 95% Confidence Interval (CI). The meta-analytic mean effect across all study units (black diamond) was calculated without any moderator terms. Arrows on error bars indicate that the CI exceeds the provided x-axis scale. Study unit ID numbers correspond to Table 1. Heterogeneity test statistic (Q), degrees of freedom (df), and *p*-value is reported for Cochran's Q test of heterogeneity for the overall model.

In the RA analysis, the intercept-only model showed no significant effect of Bd-GPL collection locality on Risk Ratio (RR = −0.996, *Z* = −0.024, *p* = 0.98; Figure 4). The RA dataset showed a high degree of heterogeneity (*I*^2^ = 95.5%; Q = 66.54, *p* = 0.001). The moderator model including life stage at exposure did not exhibit better fit than the intercept-only model (AIC = 65.06 for the full model vs. 64.55 for the intercept-only model), and reduced but did not eliminate heterogeneity (Q = 64.27, *p* < 0.001). No outliers or influential cases were detected (Cook's D < 0.45; Hat < 0.18; dfbetas < 1).

## Discussion

### Endemic Bd Lineages Are Associated With Reduced Chytridiomycosis Mortality

We performed meta-analyses of multi-strain Bd infection experiments to evaluate support for the hypotheses that the opportunity for historical or recent adaptation reduces chytridiomycosis-induced mortality. We found strong support for potential historical adaptation: we detected a significant effect of Bd genotype on Risk Ratio such that endemic Bd lineages on average resulted in reduced mortality relative to Bd-GPL, regardless of whether hosts have historically coexisted with endemic Bd lineages. This apparent difference in virulence of Bd genotypes could have resulted through a number of mechanisms, including: historical coevolution between Bd and local amphibian hosts has led to a stable state of hypovirulence in endemic Bd lineages; genomic changes have resulted in hypervirulence in Bd-GPL; and/or, as a recently emerged lineage of Bd, Bd-GPL has escaped host defense mechanisms such that even hosts that have a long co-evolutionary history with endemic Bd are functionally naïve to Bd-GPL infections. These mechanisms are not mutually exclusive. For instance, hosts that have historically coexisted with endemic Bd exhibited relatively consistent and generally low mortality rates when exposed to endemic Bd. This may indicate selection for reduced pathogen virulence and/or increased host resistance or tolerance in these systems. At the same time, ample phylogenetic and genomic evidence, the global spread of Bd-GPL, and its association with large-scale amphibian declines, support Bd-GPL as a recently emerged and widely introduced hypervirulent lineage (19, 31, 46). Independent of the mechanisms, our results confirm a strong difference in infection outcomes between endemic Bd lineages and Bd-GPL.

A notable exception to this overall pattern was Fu and Waldman (18), in which *L. caerulea* experienced high mortality from both Bd-Asia1 and Bd-GPL. The *L. caerulea* frogs used in this study were collected in New Guinea, which as of 2020 was entirely Bd-free (47). When compared with the other species in this study (*Hyla japonica, Bufo gargarizans*, and *Bombina orientalis*), which were collected from South Korea where Bd-Asia1 is historically endemic and widely distributed today (48), the pattern of disparity in outcomes due to differences in historical host adaptation opportunity is exemplified: Korean amphibians showed low mortality in both Bd-Asia1 and Bd-GPL treatments, suggesting that a long history with Bd-Asia1 may have led to evolution of broad Bd tolerance or resistance in these species. In contrast, entirely Bd-naïve *L. caerulea* frogs showed high chytridiomycosis mortality, even when infected with Bd-Asia1, which was previously reported to be hypovirulent (14). These findings suggest that Bd-Asia1 can be as virulent as Bd-GPL in naïve hosts, and that host defenses may be responsible for the apparent lack of epizootics due to Bd-Asia1 in Korean amphibian communities. Additional work is needed to assess the role of historical coexistence with endemic Bd in providing broad protection against lethal chytridiomycosis in other systems.

Individuals infected as metamorphs showed the highest Risk Ratios compared to all other life stages in the HA analysis. This may be due to the lag in the full development of the adaptive immune system following metamorphosis (49) and increased stress during this developmental transition (50, 51). Bd infects keratinized tissues in amphibians, which are restricted to mouthparts in larvae and become more widespread in post-metamorphic amphibian skin. In individuals infected as larvae, infection can shift through metamorphosis as more keratinized tissue becomes available on the body (52). Our results indicate that in the included studies, individuals infected at, rather than before, the metamorph stage, show the greatest increase in mortality due to Bd-GPL relative to endemic Bd. Metamorphosis is associated with immunosuppression, which functions to avoid a self-destructive autoimmune response as new adult-specific molecules develop but can also increase disease susceptibility in the vulnerable metamorph life stage (50). Full maturation of adult-like adaptive immunity takes a minimum of 2 months in amphibian species with fast development. Likewise, a full complement and mature levels of AMPs can be delayed until multiple months post-metamorphosis (53), although this is host species-dependent (49). Overall, our finding that individuals infected as larvae and metamorphs exhibit the highest Risk Ratios supports the idea that Bd-GPL poses a significant threat to these relatively poorly-surveyed life stages in the wild.

We note that the individuals that were infected as metamorphs in our dataset only represent a single study and species, *Bufo bufo* (14). However, this species was also exposed to comparable cumulative zoospore loads in two additional studies [57,000–136,000 zoospores over 2–4 weeks (2, 31); Table 1], and all three studies included the endemic lineage Bd-Cape. Comparing these three studies, *B. bufo* metamorphs show a higher Risk Ratio than larvae, indicating that metamorphs show the greatest difference in mortality between Bd-GPL and endemic Bd. Research on strain-specific and life stage-specific susceptibility in additional species is needed to determine whether these differences among larvae and metamorphs are common.

### Recent Coexistence Is Insufficient to Reduce Lethal Chytridiomycosis

Our Recent Adaptation analysis comparing mortality due to infection by local vs. non-local Bd-GPL showed no overall effect on disease outcomes and no significant subgroup factors. We note that there were no available studies that were performed on adults, thus interpretation of our findings should potentially be limited to larval and metamorphic life stages. The relatively high variance in the results of the RA analysis may be explained by a number of factors that can influence disease outcomes. One important factor may be the host species from which Bd strains were isolated. Due to a lack of statistical power, we were unable to test the effect of host identity from which a Bd strain was isolated. However, we note one case study with disparities based on fine-scale origin of local Bd isolate. In Waddle et al. (38), the local Bd-GPL (LBP) was isolated from a *R. onca* frog collected from the same source population used for the experiment, while the non-local Bd-GPL (LC63) was isolated from a very sick captive White's tree frog (*Litoria caerulea*). It is noteworthy that the Bd-GPL strain isolated from a stable (non-epizootic) population of the focal host resulted in functionally zero mortality (no different from control), while the Bd-GPL strain isolated from a susceptible and sick host resulted in high mortality. In another treatment, *R. onca* were exposed to a relatively local Bd-GPL (SMR) that was instead isolated from *Pseudacris regilla* located ~65 km from the nearest *R. onca* population. *P. regilla* is hypothesized to be Bd-tolerant and an important reservoir species in the western United States (54). Bd isolates from a stable population of a focal host, a susceptible species, or a reservoir species may have different consequences on the focal host as a result of selection for different virulence phenotypes. To aid in understanding the relationship between isolate origin and Bd virulence, it is important that future studies provide as much information as possible about the isolation history of Bd strains used in experiments.

In addition, local adaptation may be obscured by factors such as variation in Bd's phenotypic expression. Bd is known to exhibit phenotypic plasticity in response to temperature (55), and recent work has shown that Bd can be highly plastic over short time scales owing to functional variation in the pathogen's gene expression (56). Although amphibians can exhibit relatively fast adaptive change in immune responses (57, 58), host evolution is still expected to lag behind that of a plastic pathogen. Nonetheless, in field studies, local recovery and persistence have been documented in post-epizootic amphibian communities (59) even where local Bd pathogenicity remains high (60). Our negative RA analysis results combined with findings from wild amphibian communities suggest that recovery following Bd epizootics may not necessarily be due to short-term adaptation to local Bd strains, but rather due to broad defense against or tolerance of Bd, or avoidance of infections altogether [e.g., see (61)].

### Future Research Directions

Taken together, our results suggest that long periods of coexistence may be required for stable enzootic disease dynamics in the amphibian-chytridiomycosis system. We detected strong evidence across studies for hypovirulence in endemic Bd that may have arisen from historical coexistence with amphibian hosts. It remains unclear at what time scale adaptation of pathogen and/or host takes place, but our results do suggest that the relatively short time periods of coexistence with Bd-GPL in the geographic regions represented by the included studies (North America and Europe) have not yet been sufficient to protect many susceptible amphibian species from lethal chytridiomycosis. Further studies of the variation in disease outcomes resulting from endemic Bd lineages may provide a basis for identifying the mechanisms that reduce chytridiomycosis-induced mortality, and identifying cases where endemic Bd lineages retain virulence in naïve hosts. Similarly, host populations in which local adaptation may be beginning or in progress [e.g., in *Rana onca* (38)] may hold important insights into the potential for host adaptation to the highly successful and widely introduced Bd-GPL.

Our findings support the hypothesis that in many systems, endemic Bd lineages pose a lower threat to amphibian species than Bd-GPL. Recent studies have shown that previous infection with hypovirulent Bd-GPL strains can protect against severe chytridiomycosis due to hypervirulent Bd-GPL strains (24, 38). However, the question of whether historical coexistence with hypovirulent endemic Bd strains provide protection against Bd-GPL in the wild and/or in hosts that have not previously experienced Bd infection in their lifetimes remains to be adequately tested. Future research should focus on identifying the mechanisms that reduce lethal chytridiomycosis, which may include innate or adaptive immune mechanisms in the host, or reductions in virulence mechanisms in the pathogen. In addition, evaluating competitive dynamics between Bd-GPL and diverse endemic Bd strains may be critically important to determining the threat that Bd poses to local amphibian communities. To our knowledge, strain competition has only been assessed for Bd-GPL vs. Bd-Brazil to date (32). It remains unclear to what extent other endemic Bd lineages show similar competitive dynamics. Deepening our understanding of the relationships between co-evolutionary history, virulence, and disease outcomes will help predict and mitigate future hotspots of chytridiomycosis and aid in amphibian conservation efforts.

## Data Availability Statement

Publicly available datasets were analyzed in this study. This data can be found here: https://github.com/m-bletz/Frontiers-meta-analysis-2021.

## Author Contributions

AB, MB, IR, and KZ conceived of the study. AB, IR, and MB compiled the data and created the figures. MB performed the analyses and visualized results. AB led manuscript writing. AB and MB co-led revisions. All authors contributed to writing and revising the manuscript.

## Funding

This work was funded by a David H. Smith Postdoctoral Research Fellowship and a National Science Foundation Postdoctoral Research Fellowship in Biology that supported AB and NSF Career Grant to Douglas Woodhams that supported MB.

## Conflict of Interest

The authors declare that the research was conducted in the absence of any commercial or financial relationships that could be construed as a potential conflict of interest.

## Publisher's Note

All claims expressed in this article are solely those of the authors and do not necessarily represent those of their affiliated organizations, or those of the publisher, the editors and the reviewers. Any product that may be evaluated in this article, or claim that may be made by its manufacturer, is not guaranteed or endorsed by the publisher.

## References

[1] Fisher MC Henk DA Briggs CJ Brownstein JS Madoff LC McCraw SL. Emerging fungal threats to animal, plant and ecosystem health. Nature. (2012) 484:186–94. 10.1038/nature10947 22498624 PMC3821985

[2] Fisher MC Bosch J Yin Z Stead DA Walker J Selway L. Proteomic and phenotypic profiling of the amphibian pathogen Batrachochytrium dendrobatidis shows that genotype is linked to virulence. Mol Ecol. (2009) 18:415–29. 10.1111/j.1365-294X.2008.04041.x 19161465

[3] Lambertini C Becker CG Jenkinson TS Rodriguez D da Silva Leite D James TY. Local phenotypic variation in amphibian-killing fungus predicts infection dynamics. Fungal Ecol. (2016) 20:15–21. 10.1016/j.funeco.2015.09.014

[4] Becker CG Greenspan SE Tracy KE Dash JA Lambertini C Jenkinson TS. Variation in phenotype and virulence among enzootic and panzootic amphibian chytrid lineages. Fungal Ecol. (2017) 26:45–50. 10.1016/j.funeco.2016.11.007

[5] Adams AJ Kupferberg SJ Wilber MQ Pessier AP Grefsrud M Bobzien S. Extreme drought, host density, sex, and bullfrogs influence fungal pathogen infection in a declining lotic amphibian. Ecosphere. (2017) 8:e01740. 10.1002/ecs2.1740

[6] Turner A Wassens S Heard G Peters A. Temperature as a driver of the pathogenicity and virulence of amphibian chytrid fungus Batrachochytrium dendrobatidis: a systematic review. J Wildl Dis. (2021) 57:477–94. 10.7589/JWD-D-20-00105 34019674

[7] May RM Anderson RM Bodmer WF Kingman JFC. Epidemiology and genetics in the coevolution of parasites and hosts. Proc R Soc Lond B Biol Sci. (1983) 219:281–313. 10.1098/rspb.1983.0075 6139816

[8] Retallick RWR McCallum H Speare R. Endemic infection of the Amphibian Chytrid Fungus in a frog community post-decline. PLoS Biol. (2004) 2:e351. 10.1371/journal.pbio.0020351 15502873 PMC521176

[9] Savage AE Sredl MJ Zamudio KR. Disease dynamics vary spatially and temporally in a North American amphibian. Biol Conserv. (2011) 144:1910–5. 10.1016/j.biocon.2011.03.018

[10] Thompson JN. Specific hypotheses on the geographic mosaic of coevolution. Am Nat. (1999) 153:S1–14. 10.1086/30320833352070

[11] Wake DB Vredenburg VT. Are we in the midst of the sixth mass extinction? A view from the world of amphibians. Proc Natl Acad Sci USA. (2008) 105:11466–73. 10.1073/pnas.0801921105 18695221 PMC2556420

[12] Scheele BC Pasmans F Skerratt LF Berger L Martel A Beukema W. Amphibian fungal panzootic causes catastrophic and ongoing loss of biodiversity. Science. (2019) 363:1459–63. 10.1126/science.aav0379 30923224

[13] Lambert MR Womack MC Byrne AQ Hernández-Gómez O Noss CF Rothstein AP. Comment on “Amphibian fungal panzootic causes catastrophic and ongoing loss of biodiversity.” *Science*. (2020) 367:eaay1838. 10.1126/science.aay1838 32193293

[14] O'Hanlon SJ Rieux A Farrer RA Rosa GM Waldman B Bataille A. Recent Asian origin of chytrid fungi causing global amphibian declines. Science. (2018) 360:621–7. 10.1126/science.aar1965 29748278 PMC6311102

[15] Schloegel LLM Toledo LFL Longcore JE Greenspan SE Vieira CA Lee M. Novel, panzootic and hybrid genotypes of amphibian chytridiomycosis associated with the bullfrog trade. Mol Ecol. (2012) 21:5162–77. 10.1111/j.1365-294X.2012.05710.x 22857789

[16] James TY Toledo LF Rödder D da Silva Leite D Belasen AM Betancourt-Román CM. Disentangling host, pathogen, and environmental determinants of a recently emerged wildlife disease: Lessons from the first 15 years of amphibian chytridiomycosis research. Ecol Evol. (2015) 5:4079–97. 10.1002/ece3.1672 26445660 PMC4588650

[17] Rachowicz LJ Hero J-M Alford RA Taylor JW Morgan JAT Vredenburg VT. The novel and endemic pathogen hypotheses: competing explanations for the origin of emerging infectious diseases of wildlife. Conserv Biol. (2005) 19:1441–8. 10.1111/j.1523-1739.2005.00255.x

[18] Fu M Waldman B. Ancestral chytrid pathogen remains hypervirulent following its long coevolution with amphibian hosts. Proc R Soc B Biol Sci. (2019) 286:20190833. 10.1098/rspb.2019.0833 31161901 PMC6571470

[19] Farrer RA Martel A Verbrugghe E Abouelleil A Ducatelle R Longcore JE. Genomic innovations linked to infection strategies across emerging pathogenic chytrid fungi. Nat Commun. (2017) 8:14742. 10.1038/ncomms14742 28322291 PMC5364385

[20] Rodriguez D Becker CG Pupin NC Haddad CFB Zamudio KR. Long-term endemism of two highly divergent lineages of the amphibian-killing fungus in the Atlantic Forest of Brazil. Mol Ecol. (2014) 23:774–87. 10.1111/mec.12615 24471406

[21] Talley BL Muletz CR Vredenburg VT Fleischer RC Lips KR. A century of *Batrachochytrium dendrobatidis* in Illinois amphibians (1888–1989). Biol Conserv. (2015) 182:254–61. 10.1016/j.biocon.2014.12.007

[22] Adams AJ Pessier AP Briggs CJ. Rapid extirpation of a North American frog coincides with an increase in fungal pathogen prevalence: historical analysis and implications for reintroduction. Ecol Evol. (2017) 7:10216–32. 10.1002/ece3.3468 29238549 PMC5723621

[23] Jenkinson TS Román CMB Lambertini C Valencia-Aguilar A Rodriguez D Nunes-de-Almeida CHL. Amphibian-killing chytrid in Brazil comprises both locally endemic and globally expanding populations. Mol Ecol. (2016) 25:2978–96. 10.1111/mec.13599 26939017

[24] Greener MS Verbrugghe E Kelly M Blooi M Beukema W Canessa S. Presence of low virulence chytrid fungi could protect European amphibians from more deadly strains. Nat Commun. (2020) 11:5393. 10.1038/s41467-020-19241-7 33106491 PMC7589487

[25] Yap TA Koo MS Ambrose RF Vredenburg VT. Introduced bullfrog facilitates pathogen invasion in the western United States. PLoS ONE. (2018) 13:e0188384. 10.1371/journal.pone.0188384 29659568 PMC5901863

[26] Blaustein AR Urbina J Snyder PW Reynolds E Dang T Hoverman JT. Effects of emerging infectious diseases on amphibians: a review of experimental studies. Diversity. (2018) 10:81. 10.3390/d10030081

[27] Harzing A. Publish or Perish. (2007). Available online at: http://www.harzing.com/pop.htm

[28] McDonald CA. Too Little, Too Late: Amphibian Responses to Chytrid Fungus Across Time and Space (2021).

[29] Doddington BJ Bosch J Oliver JA Grassly NC Garcia G Schmidt BR. Context-dependent amphibian host population response to an invading pathogen. Ecology. (2013) 94:1795–804. 10.1890/12-1270.1 24015523

[30] Greenspan SE Lambertini C Carvalho T James TY Toledo LF Haddad CFB. Hybrids of amphibian chytrid show high virulence in native hosts. Sci Rep. (2018) 8:9600. 10.1038/s41598-018-27828-w 29941894 PMC6018099

[31] Farrer RA Weinert LA Bielby J Garner TWJ Balloux F Clare F. Multiple emergences of genetically diverse amphibian-infecting chytrids include a globalized hypervirulent recombinant lineage. Proc Natl Acad Sci USA. (2011) 108:18732–6. 10.1073/pnas.1111915108 22065772 PMC3219125

[32] Jenkinson TS Rodriguez D Clemons RA Michelotti LA Zamudio KR Toledo LF. Globally invasive genotypes of the amphibian chytrid outcompete an enzootic lineage in coinfections. Proc R Soc B. (2018) 285:20181894. 10.1098/rspb.2018.1894 30963903 PMC6304064

[33] Burrow AK Rumschlag SL Boone MD. Host size influences the effects of four isolates of an amphibian chytrid fungus. Ecol Evol. (2017) 7:9196–202. 10.1002/ece3.3255 29187961 PMC5696404

[34] Gahl MK Longcore JE Houlahan JE. Varying responses of Northeastern North American Amphibians to the Chytrid Pathogen Batrachochytrium dendrobatidis. Conserv Biol. (2012) 26:135–41. 10.1111/j.1523-1739.2011.01801.x 22181933

[35] Dang TD Searle CL Blaustein AR. Virulence variation among strains of the emerging infectious fungus *Batrachochytrium dendrobatidis* (Bd) in multiple amphibian host species. Dis Aquat Organ. (2017) 124:233–9. 10.3354/dao03125 28492179

[36] Meurling S Cortazar-Chinarro M Siljestam M Åhlen D Ågren E Höglund J. Body size mediates latitudinal population differences in response to Bd infection in two amphibian species. (2021). 1–36. 10.1101/2021.07.16.452656 PMC10830819 38097779

[37] Arellano ML Natale GS Grilli PG Barrasso DA Steciow MM Lavilla EO. Host-pathogen relationships between the chytrid fungus Batrachochytrium dendrobatidis and tadpoles of five South American anuran species. Herpetol J. 27:33–39. Available online at: http://sedici.unlp.edu.ar/handle/10915/118298

[38] Waddle AW Levy JE Rivera R van Breukelen F Nash M Jaeger JR. Population-level resistance to Chytridiomycosis is life-stage dependent in an Imperiled Anuran. EcoHealth. (2019) 16:701–11. 10.1007/s10393-019-01446-y 31654279

[39] R Core Team. R: A Language and Environment for Statistical Computing. Vienna: R Foundation of Statistical Computing (2021).

[40] Pick JL Nakagawa S Noble DWA. Reproducible, flexible and high-throughput data extraction from primary literature: the METADIGITISE R package. Methods Ecol Evol. (2019) 10:426–31. 10.1111/2041-210X.13118

[41] Viechtbauer W. Conducting Meta-Analyses in R with the metafor Package. J Stat Softw. (2010) 36:1–48. 10.18637/jss.v036.i03

[42] Higgins JPT Thompson SG. Quantifying heterogeneity in a meta-analysis. Stat Med. (2002) 21:1539–58. 10.1002/sim.1186 12111919

[43] Harrer M Cuijpers P A FT Ebert DD. Doing Meta-Analysis With R: A Hands-On Guide. 1st ed. Boca Raton, FL and London: Chapman & Hall/CRC Press (2021). 10.1201/9781003107347

[44] Noble DWA Lagisz M O'dea RE Nakagawa S. Non-independence and sensitivity analyses in ecological and evolutionary meta-analyses. Mol Ecol. (2017) 26:2410–25. 10.1111/mec.14031 28133832

[45] Wickham H. ggplot2: Elegant Graphics for Data Analysis. New York, NY: Springer (2009). 10.1007/978-0-387-98141-3

[46] James TY Litvintseva AP Vilgalys R Morgan JA Taylor JW Fisher MC. Rapid global expansion of the fungal disease chytridiomycosis into declining and healthy amphibian populations. PLoS Pathog. (2009) 5:e1000458. 10.1371/journal.ppat.1000458 19478871 PMC2680619

[47] Bower DS Jennings CK Webb RJ Amepou Y Schwarzkopf L Berger L. Disease surveillance of the amphibian chytrid fungus *Batrachochytrium dendrobatidis* in Papua New Guinea. Conserv Sci Pract. (2020) 2:e256. 10.1111/csp2.256

[48] Bataille A Fong JJ Cha M Wogan GOU Baek HJ Lee H. Genetic evidence for a high diversity and wide distribution of endemic strains of the pathogenic chytrid fungus *Batrachochytrium dendrobatidis* in wild Asian amphibians. Mol Ecol. (2013) 22:4196–209. 10.1111/mec.12385 23802586

[49] Rollins-Smith LA. Amphibian immunity–stress, disease, and climate change. Dev Comp Immunol. (2017) 66:111–9. 10.1016/j.dci.2016.07.002 27387153

[50] Rollins-Smith LA. Metamorphosis and the amphibian immune system. Immunol Rev. (1998) 166:221–30. 10.1111/j.1600-065X.1998.tb01265.x 9914915

[51] Robert J Ohta Y. Comparative and developmental study of the immune system in Xenopus. Dev Dyn Off Publ Am Assoc Anat. (2009) 238:1249–70. 10.1002/dvdy.21891 19253402 PMC2892269

[52] Marantelli G Berger L Speare R Keegan L. Distribution of the amphibian chytrid Batrachochytrium dendrobatidis and keratin during tadpole development. Pac Conserv Biol. (2004) 10:173. 10.1071/PC040173

[53] Holden WM Hanlon SM Woodhams DC Chappell TM Wells HL Glisson SM. Skin bacteria provide early protection for newly metamorphosed southern leopard frogs (Rana sphenocephala) against the frog-killing fungus, *Batrachochytrium dendrobatidis*. Biol Conserv. (2015) 187:91–102. 10.1016/j.biocon.2015.04.007

[54] Reeder NMM Pessier AP Vredenburg VT. A reservoir species for the emerging amphibian pathogen Batrachochytrium dendrobatidis thrives in a landscape decimated by disease. PLoS ONE. (2012) 7:e33567. 10.1371/journal.pone.0033567 22428071 PMC3299797

[55] Muletz-Wolz CR Barnett SE DiRenzo GV Zamudio KR Toledo LF James TY. Diverse genotypes of the amphibian-killing fungus produce distinct phenotypes through plastic responses to temperature. J Evol Biol. (2019) 32:287–98. 10.1111/jeb.13413 30650220

[56] McDonald CA Ellison AR Toledo LF James TY Zamudio KR. Gene expression varies within and between enzootic and epizootic lineages of Batrachochytrium dendrobatidis (Bd) in the Americas. Fungal Biol. (2020) 124:34–43. 10.1016/j.funbio.2019.10.008 31892375

[57] Savage AE Zamudio KR. MHC genotypes associate with resistance to a frog-killing fungus. Proc Natl Acad Sci USA. (2011) 108:16705–10. 10.1073/pnas.1106893108 21949385 PMC3189034

[58] Bataille A Cashins SD Grogan L Skerratt LF Hunter D McFadden M. Susceptibility of amphibians to chytridiomycosis is associated with MHC class II conformation. Proc Biol Sci. (2015) 282:20143127. 10.1098/rspb.2014.3127 25808889 PMC4389617

[59] Scheele BC Skerratt LF Grogan LF Hunter DA Clemann N McFadden M. After the epidemic: ongoing declines, stabilizations and recoveries in amphibians afflicted by chytridiomycosis. Biol Conserv. (2017) 206:37–46. 10.1016/j.biocon.2016.12.010

[60] Voyles J Woodhams DC Saenz V Byrne AQ Perez R Rios-Sotelo G. Shifts in disease dynamics in a tropical amphibian assemblage are not due to pathogen attenuation. Science. (2018) 359:1517–9. 10.1126/science.aao4806 29599242

[61] Bell SC Heard GW Berger L Skerratt LF. Connectivity over a disease risk gradient enables recovery of rainforest frogs. Ecol Appl. (2020) 30:e02152. 10.1002/eap.2152 32343856

