# Supplementary material for: Endemic Lineages of Batrachochytrium dendrobatidis Are Associated With Reduced Chytridiomycosis-Induced Mortality in Amphibians: Evidence From a Meta-Analysis of Experimental Infection Studies
Source: Front Vet Sci. 2022 Mar 4;9:756686. doi: 10.3389/fvets.2022.756686 (PMC8931402; doi:10.3389/fvets.2022.756686)
Supplement: Supplementary file 1 [file Image_1.pdf]

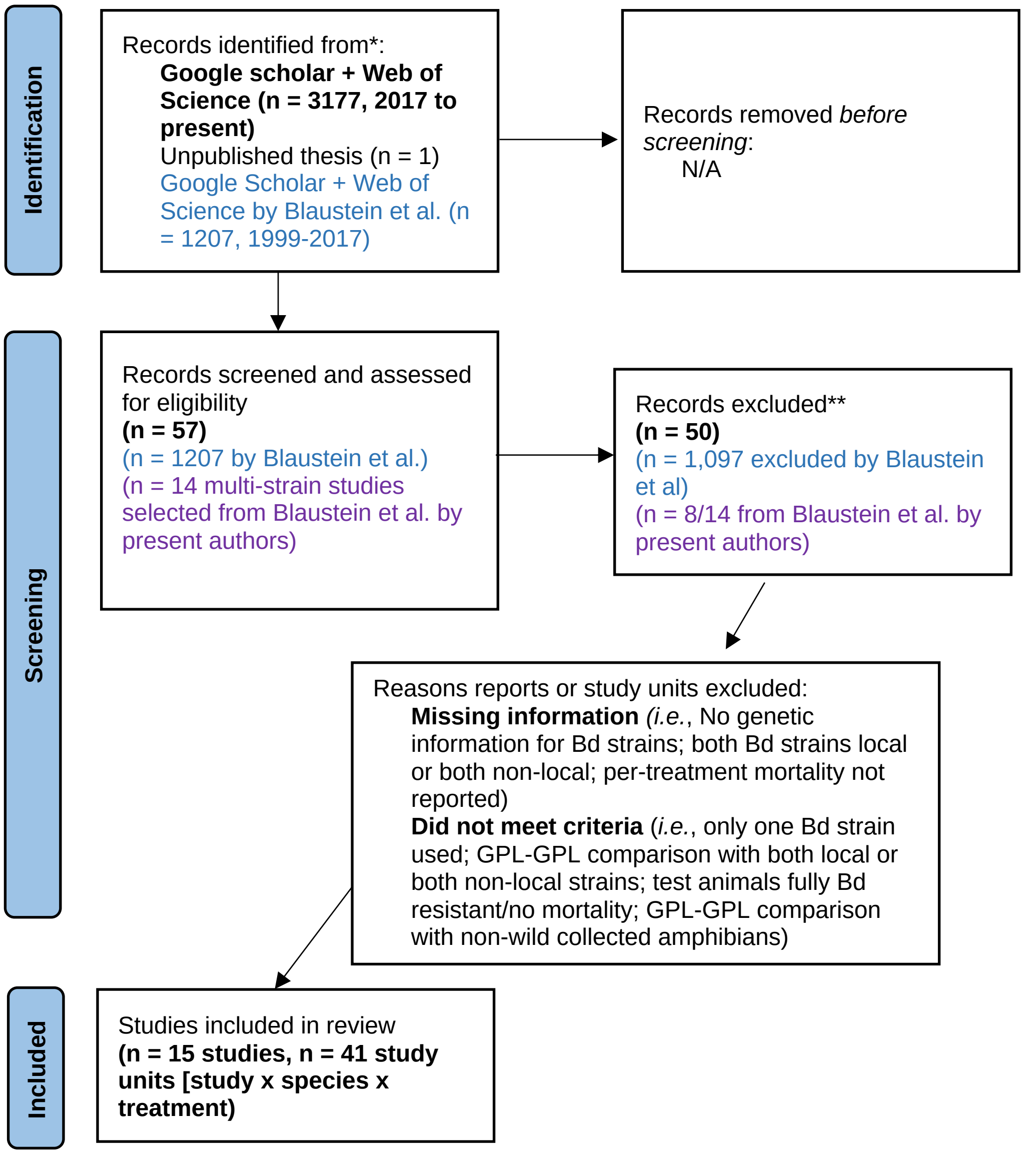

PRISMA diagram citation: Page MJ, McKenzie JE, Bossuyt PM, Boutron I, Hoffmann TC, Mulrow CD, et al. The PRISMA 2020 statement: an updated guideline for reporting systematic reviews. BMJ 2021;372:n71. doi: 10.1136/bmj.n71

Figure S1. PRISMA diagram depicting results of literature search and filtering process.
