# Supplementary figures and images for: Endemic Lineages of Batrachochytrium dendrobatidis Are Associated With Reduced Chytridiomycosis-Induced Mortality in Amphibians: Evidence From a Meta-Analysis of Experimental Infection Studies
Source: Front Vet Sci. 2022 Mar 4;9:756686. doi: 10.3389/fvets.2022.756686 (PMC8931402; doi:10.3389/fvets.2022.756686)

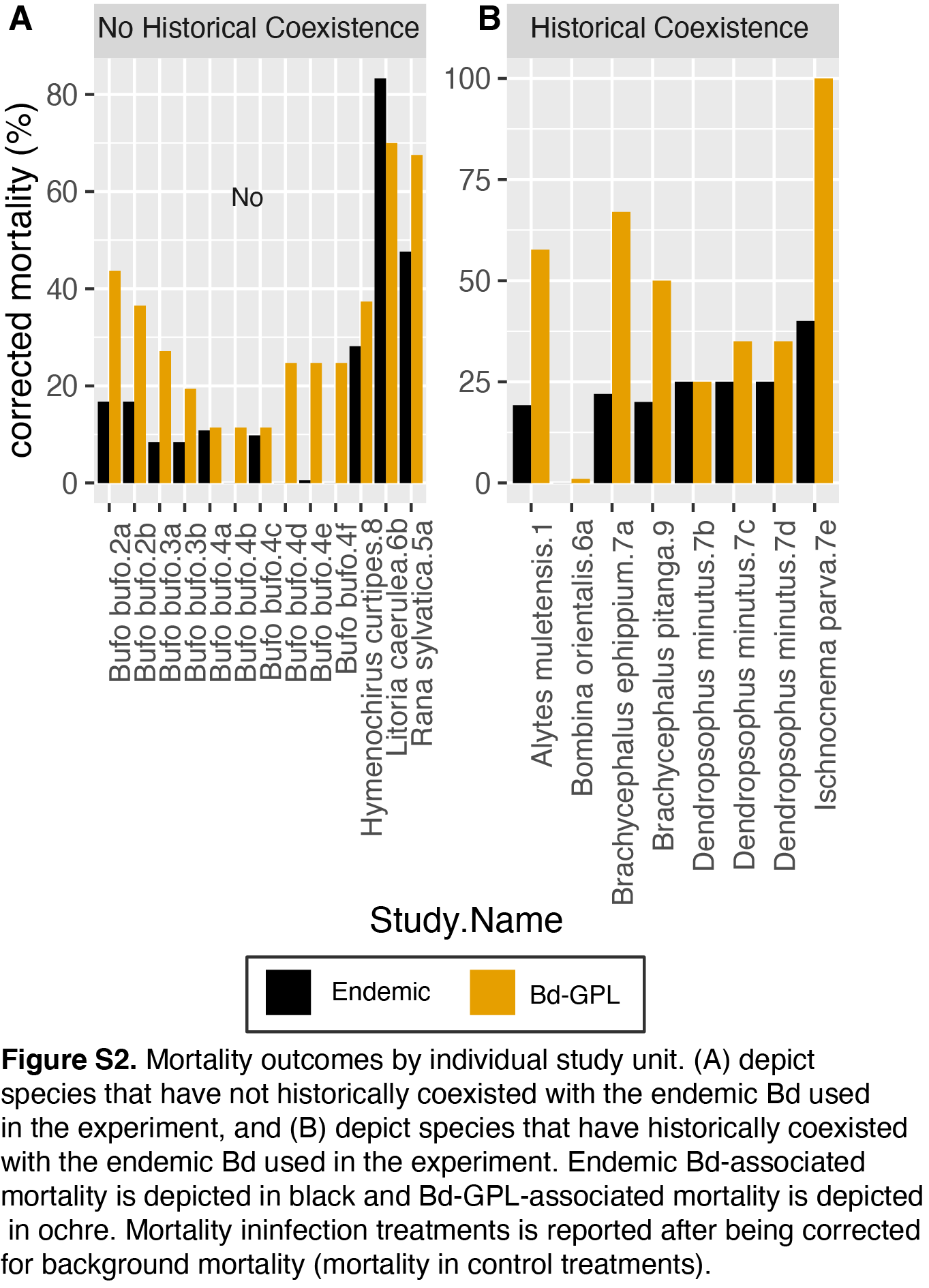

Supplement: Supplementary file 2 [file Image_2.tif]
