# Supplementary material for: Endemic Lineages of Batrachochytrium dendrobatidis Are Associated With Reduced Chytridiomycosis-Induced Mortality in Amphibians: Evidence From a Meta-Analysis of Experimental Infection Studies
Source: Front Vet Sci. 2022 Mar 4;9:756686. doi: 10.3389/fvets.2022.756686 (PMC8931402; doi:10.3389/fvets.2022.756686)
Supplement: Supplementary file 3 [file Table_1.DOCX]

**Table S1.** Table S1. Genotyping method information for Bd strains used in included experiments. Methods abbreviations ae as follows: MLST = multi-locus sequence typing, WGS = whole genome sequencing, FA = fluidigm array. References followed by numbers are present in full in the main document reference list. Additional references are marked with an asterisk (*); full citations are below the table.

|  | **Host species** | **Study** | ***Study unit number*** | **Endemic/local genotyping method** | **Endemic/local genotype source** | **Non-endemic/non- local genotyping method** | **Non-endemic/non-local genotype source** |
| --- | --- | --- | --- | --- | --- | --- | --- |
| **Historical Adaptation (HA) Analysis** | *Alytes muletensis* | Doddington et al. 2013 (55) | *1* | MLST and WGS | Fisher et al. 2009 (2) Rosenblum et al. 2013* O'Hanlon et al. 2018 (14) | MLST and WGS | Fisher et al. 2009 (2) Rosenblum et al. 2013* O'Hanlon et al. 2018 (14) |
|  | *Bombina orientalis* | Fu and Waldman 2019 (18) | *6a* | WGS | O'Hanlon et al. 2018 (14) | WGS and FA | Rosenblum et al. 2013* O'Hanlon et al. 2018 (14) Byrne et al. 2019* |
|  | *Brachycephalus ephippium* | Greenspan et al. 2018 (56) | *7a* | MLST | Greenspan et al. 2018 (56) | MLST | Greenspan et al. 2018 (56) |
|  | *Brachycephalus pitanga* | McDonald 2021 (28) | *9* | WGS and FA | Rosenblum et al. 2013* O'Hanlon et al. 2018 (14) Byrne et al. 2019* | MLST | Greenspan et al. 2018 (56) |
|  | *Bufo bufo* | Fisher et al. 2009 (2) | *2a-b* | MLST and WGS | Fisher et al. 2009 Farrer et al. 2011 O'Hanlon et al. 2018 | MLST and WGS | Fisher et al. 2009 (2) Rosenblum et al. 2013* O'Hanlon et al. 2018 (14) |
|  |  | Farrer et al. 2011 (37) | *3a-b* | MLST and WGS | Fisher et al. 2009 (2) Rosenblum et al. 2013* O'Hanlon et al. 2018 (14) | MLST and WGS | Fisher et al. 2009 (2) Rosenblum et al. 2013* O'Hanlon et al. 2018 (14) |
|  |  | O'Hanlon et al. 2018 (14) | *4a-f* | WGS | O'Hanlon et al. 2018 (14) | WGS | O'Hanlon et al. 2018 (14) |
|  | *Dendropsophus minutus* | Greenspan et al. 2018 (56) | *7b* | MLST | Greenspan et al. 2018 (56) | MLST | Greenspan et al. 2018 (56) |
|  | *Hymenochirus curtipes* | Jenkinson et al. 2018 (54) | *8* | MLST | Greenspan et al. 2018 (56) | MLST | Greenspan et al. 2018 (56) |
|  | *Ischnocnema parva* | Greenspan et al. 2018 (56) | *7c* | MLST | Greenspan et al. 2018 (56) | MLST | Greenspan et al. 2018 (56) |
|  | *Rana sylvatica* | Becker et al. 2017 (4) | *5a* | MLST and WGS | Rosenblum et al. 2013* Schloegel et al. 2012 (15) O'Hanlon et al. 2018 (14) Byrne et al. 2019* | MLST | Schloegel et al. 2010* |
|  | *Litoria caerulea* | Fu and Waldman 2019 (18) | *6b* | WGS | O'Hanlon et al. 2018 (14) | WGS and FA | Rosenblum et al. 2013* O'Hanlon et al. 2018 (14) Byrne et al. 2019* |
| **Recent Adaptation (RA) Analysis** | *Alytes obstetricans* | Greener et al. 2020 (24) | *10a-d* | WGS and FA | O'Hanlon et al. 2018 (14) Byrne et al. 2019* | MLST and WGS | Farrer et al. 2011 (37) Rosenblum et al 2013 Schloegel et al. 2012 (15) O'Hanlon et al. 2018 (14) |
|  | *Anaxyrus americanus* | Burrow et al. 2017 (57) | *11a-c* | MLST | Schloegel et al. 2012 (15) | MLST and WGS | Farrer et al. 2011 (37) Rosenblum et al 2013 Schloegel et al. 2012 (15) O'Hanlon et al. 2018 (14) |
|  | *Anaxyrus americanus* | Gahl et al. 2012 (58) | *12a* | MLST | Schloegel et al. 2012 (15) | MLST and WGS | Farrer et al. 2011 (37) Rosenblum et al 2013 Schloegel et al. 2012 (15) O'Hanlon et al. 2018 (14) |
|  | *Anaxyrus boreas* | Dang et al. 2017 (59) | *13a-b* | MLST and WGS | Schloegel et al. 2012 (15) Rosenblum et al. 2013* | MLST and WGS | Schloegel et al. 2012 (15) Rosenblum et al. 2013* |
|  | *Bufo bufo* | Fisher et al. 2009 (2) | *2c* | MLST and WGS | Fisher et al. 2009 (2) Rosenblum et al. 2013* O'Hanlon et al. 2018 (14) | MLST and WGS | Fisher et al. 2009 (2) Farrer et al. 2011 (37) |
|  |  | Meurling et al. 2021(60) | *14* | WGS | O'Hanlon et al. 2018 (14) | WGS and FA | O'Hanlon et al. 2018 (14) Byrne et al. 2019* |
|  | *Physalaemus fernandezae* | Arellano et al. 2017 (61) | *15* | WGS | O'Hanlon et al. 2018 (14) | MLST and WGS | Farrer et al. 2011 (37) Rosenblum et al 2013 Schloegel et al. 2012 (15) O'Hanlon et al. 2018 (14) |
|  | *Pseudacris regilla* | Dang et al. 2017 (59) | *13c-d* | MLST and WGS | Schloegel et al. 2012 (15) Rosenblum et al. 2013* | MLST and WGS | Schloegel et al. 2012 Rosenblum et al. 2013* |
|  | *Rana clamitans* | Gahl et al. 2012 (58) | *12b* | MLST | Schloegel et al. 2010* | MLST and WGS | Farrer et al. 2011 (37) Rosenblum et al 2013 Schloegel et al. 2012 (15) O'Hanlon et al. 2018 (14) |
|  | *Rana pipiens* | Gahl et al. 2012 (58) | *12c* | MLST | Schloegel et al. 2010* | MLST and WGS | Farrer et al. 2011 (37) Rosenblum et al 2013 Schloegel et al. 2012 (15) O'Hanlon et al. 2018 (14) |
|  | *Rana sylvatica* | Gahl et al. 2012 (58) | *12d* | MLST | Schloegel et al. 2010* | MLST and WGS | Farrer et al. 2011 (37) Rosenblum et al 2013 Schloegel et al. 2012 (15) O'Hanlon et al. 2018 (14) |
|  |  | Becker et al. 2017 (4) | *5b* | MLST | Schloegel et al. 2012 (15) | MLST and WGS | Farrer et al. 2011 (37) Rosenblum et al 2013 Schloegel et al. 2012 (15) O'Hanlon et al. 2018 (14) |
|  | *Rana cascadae* | Dang et al. 2017 (59) | *13e-f* | MLST and WGS | Schloegel et al. 2012 (15) Rosenblum et al. 2013* | MLST and WGS | Schloegel et al. 2012 (15) Rosenblum et al. 2013* |
|  | *Rana onca* | Waddle et al. 2019 (29) | *16a-b* | FA | Byrne et al. 2019* | none | Inferred by current authors - isolated from captive Australian frog |

*Additional references:

Byrne, A. Q., et al. "Cryptic diversity of a widespread global pathogen reveals expanded threats to amphibian conservation." Proceedings of the National Academy of Sciences 116.41 (2019): 20382-20387.

Rosenblum, E. B., et al. 2013. "Complex history of the amphibian-killing chytrid fungus revealed with genome resequencing data." Proceedings of the National Academy of Sciences 110.23: 9385-9390.

Schloegel, L. M., et al. "The North American bullfrog as a reservoir for the spread of Batrachochytrium dendrobatidis in Brazil." Animal Conservation 13 (2010): 53-61.
